# WNT16 Overexpression is Insufficient to Counteract Inflammation-induced Bone Loss in Female Mice

**DOI:** 10.1007/s00223-026-01481-2

**Published:** 2026-01-31

**Authors:** Karin H. Nilsson, Petra Henning, Marie K. Lagerquist, Jianyao Wu, Marta Bally, Ulf H. Lerner, Inger Gjertsson, Claes Ohlsson, Sofia Movérare-Skrtic

**Affiliations:** 1 Department of Internal Medicine and Clinical Nutrition, Sahlgrenska Osteoporosis Centre, Centre for Bone and Arthritis Research at the Sahlgrenska Academy, Institute of Medicine, University of Gothenburg, Gothenburg, Sweden; 2 Division of Biological Physics, Department of Applied Physics, Chalmers University of Technology, Gothenburg, Sweden; 3 Department of Rheumatology and Inflammation Research, Institute of Medicine, University of Gothenburg, Gothenburg, Sweden; 4 Department of Rheumatology, Sahlgrenska University Hospital, Gothenburg, Sweden; 5 Unit of Clinical Pharmacology, Department of Pharmaceuticals, Sahlgrenska University Hospital, Region Västra Götaland, Gothenburg, Sweden; 6 Present Address: Department of Clinical Microbiology , Umeå University, Umeå, Sweden; 7 Present Address: Wallenberg Centre for Molecular Medicine, Umeå University, Umeå, Sweden

**Keywords:** Osteoporosis, Inflammation, Wnt signaling

## Abstract

Osteoporosis is characterized by an imbalance in bone remodeling, resulting in bone loss and increased fracture risk. Inflammatory diseases, such as rheumatoid arthritis, are strongly associated with secondary osteoporosis due to inflammation-induced bone loss. Pro-inflammatory cytokines, particularly TNF-α, disrupt bone homeostasis by promoting osteoclastogenesis and inhibiting osteoblast function. The Wnt signaling pathway is essential for bone formation and is suppressed in inflammatory conditions. WNT16, an osteoblast-derived ligand, increases bone mass mainly by inhibiting osteoclast differentiation but has also been found to stimulate osteoblast activity. Here we demonstrate that TNF-α downregulates *Wnt16* mRNA expression in primary osteoblasts, suggesting that inflammation may impair WNT16 expression and thereby reduce bone mass. To evaluate whether pharmacological or genetical elevation of WNT16 levels can mitigate inflammation-induced bone loss, we examined the effect of WNT16 in three mouse models of local and systemic inflammation. In a knee arthritis model, intra-articular delivery of WNT16 liposomes failed to prevent local bone loss. Similarly, although osteoblast-specific WNT16 overexpression increased the overall bone mass, it did not protect against either local calvarial bone loss or systemic bone loss induced by Toll-like receptor 2 (TLR2) activation. Furthermore, in a model of systemic inflammation induced by *Staphylococcus aureus*, WNT16 overexpression did not preserve vertebral trabecular bone, despite increased baseline bone mass. These findings demonstrate that WNT16, although increasing the overall bone mass, is insufficient to counteract inflammation-driven bone loss.

## Introduction

Osteoporosis is a common systemic skeletal disease characterized by an unbalanced bone-remodelling activity leading to bone loss, deterioration of bone microarchitecture, and increased risk of fractures. In secondary osteoporosis, low bone mass is caused by underlying diseases or medications (reviewed in [1]). Epidemiologic studies have shown a higher prevalence of osteoporosis in patients with autoimmune and chronic inflammatory diseases, such as rheumatoid arthritis (RA) and inflammatory bowel diseases [2]. In RA, the risk of both hip and vertebral fractures is approximately doubled compared to age and gender-matched controls [3–5], highlighting inflammation-mediated bone loss as a major clinical problem.

Bone homeostasis depends on the balance between bone resorption by osteoclasts and bone formation by osteoblasts. In addition to building bone, osteoblasts regulate osteoclast differentiation through the expression of stimulatory and inhibitory factors, such as RANKL inducing osteoclast differentiation and osteoprotegerin (OPG), which suppresses osteoclastogenesis. Chronic inflammation, as seen in RA, disrupts this balance, leading to both local and systemic bone loss [2]. This is primarily driven by the production and release of pro-inflammatory cytokines, especially tumor necrosis factor-alpha (TNF-α), in response to autoantibodies [6]. TNF-α promotes osteoclast differentiation and impairs osteoblast function, thereby skewing the remodeling balance in favor of resorption [7, 8]. Although the impact of TNF-α on inflammation-induced bone loss is well-established, the precise molecular mechanisms mediating this effect are not yet fully understood.

The Wnt signaling pathway is essential for regulating cell proliferation, differentiation, and tissue homeostasis, with a particular important role in osteoblast differentiation [9]. In inflammatory diseases, this pathway is downregulated through the induction of Dickkopf-related protein 1 (DKK1), a Wnt signaling pathway inhibitor, resulting in reduced bone formation [10, 11]. Recent findings from our group identified WNT16, an osteoblast-derived ligand, as a positive regulator of bone mass [12, 13]. WNT16 suppresses osteoclast differentiation via paracrine signaling and enhances osteoblast activity through autocrine/paracrine effects. Although WNT16 increases overall bone mass, it remains unclear whether pharmacological or genetical elevation of WNT16 levels can specifically inhibit inflammation-induced bone loss.

In this study, we investigated the potential protective role of enhanced WNT16 levels in inflammation-induced bone loss using three mouse models: (i) a model of local bone loss in the knee joint [14], (ii) a model combining local and systemic inflammation [15], and (iii) a model of *Staphylococcus aureus*-induced systemic bone loss [16]. To explore the effects of enhanced WNT16 levels, we used both pharmacological treatment, previously shown to increase bone mass [12], and genetic overexpression of WNT16 [13]. By employing these approaches, we aimed to elucidate the potential protective role of elevated WNT16 levels in mitigating inflammation-induced bone loss.

## Materials and Methods

### Animal Experiments

The experimental procedures involving animals were approved by the Ethics Committee in Gothenburg, Västra Götaland, or Umeå University, and all methods were carried out in accordance with relevant guidelines and regulations and in accordance with ARRIVE guidelines. Female mice were used to ensure consistency across the different models and because osteoporosis-related fractures occur more often in women than in men. The mice were housed in a standard animal housing facility with 12-h of darkness and 12-h of light photo period. The temperature was controlled (22 °C) and food and water were available ad libitum.

### Mouse Model of Antigen-Induced Arthritis (AIA)

**Fig. 1:**
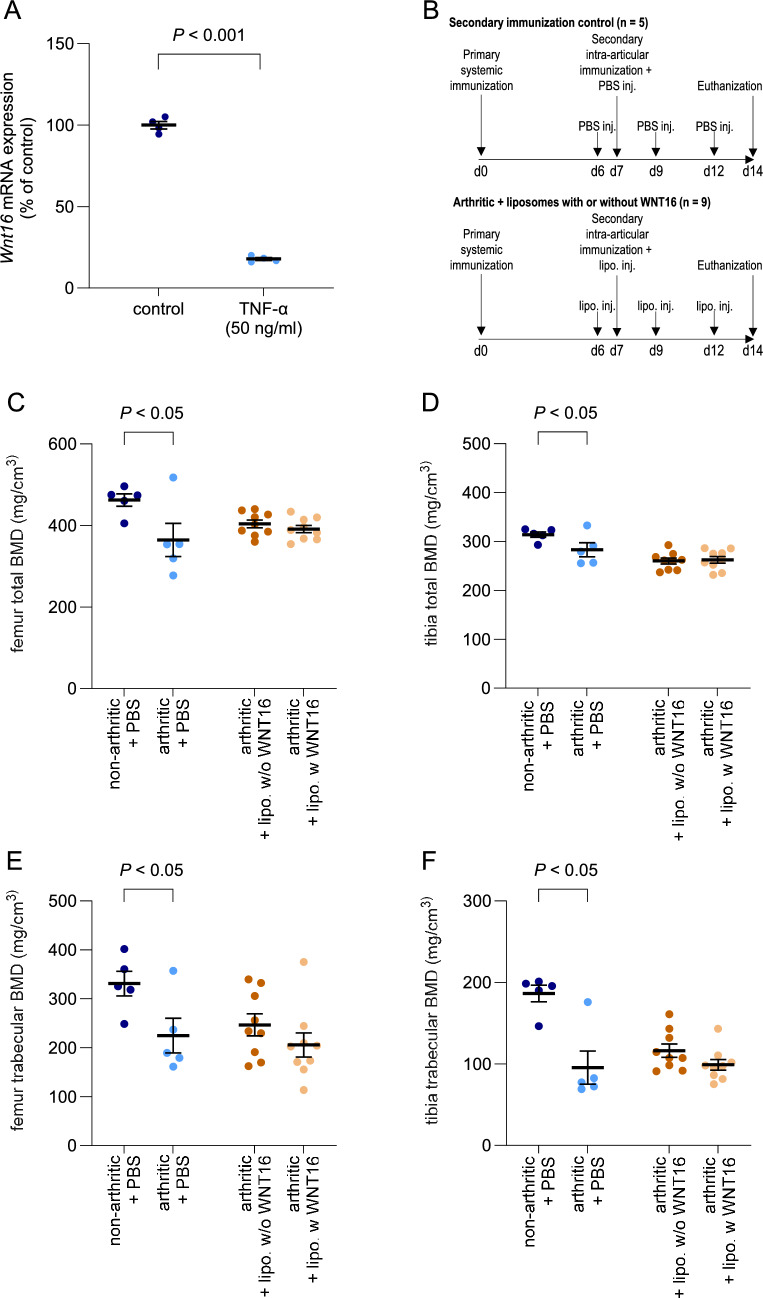
TNF-α reduces *Wnt16* mRNA expression in osteoblasts, but WNT16 treatment does not prevent periarticular bone loss in antigen-induced arthritis. **A** *Wnt16* mRNA expression in primary mouse calvarial osteoblasts cultured for 24 h in control medium with or without TNF-α (50 ng/ml). Values are presented as mean ± SEM (n = 4). Data were analyzed using an unpaired Student’s t-test. **B** Schematic representation of the antigen-induced arthritis model. Upper panel: Control group after secondary immunization (left knee = arthritic; right knee = non-arthritic). Lower panel: Liposome-treated group (left knee = arthritic + liposomes with WNT16; right knee = arthritic + empty liposomes). (C-F) Peripheral quantitative computed tomography (pQCT) analysis of bone mineral density (BMD): total BMD in the metaphyseal regions of the distal femur **C** and proximal tibia **D**, and trabecular BMD in the same regions of the distal femur **E** and proximal tibia **F**. Data were collected from bones adjacent to non-arthritic and antigen-induced arthritic mouse knees treated with PBS, as well as from bones adjacent to arthritic knees treated with liposomes with WNT16 or empty liposomes. Samples were obtained from 9-week-old female wild-type mice. Values are mean ± SEM (n = 5–9 mice per group). Data assessed by paired Student’s *t*-test. lipo. = liposomes; inj. = injection; w = with; and w/o = without

At day 0, seven-week-old wildtype (WT) C57BL/6N female littermate mice (n = 14) (Charles River, Germany) were primary immunized with 100 μl of 2 mg/ml of methylated bovine serum albumin (mBSA; Sigma-Aldrich, Stockholm, Sweden) dissolved in phosphate-buffered saline (PBS) and emulsified 1:1 in Freund’s complete adjuvant (Sigma-Aldrich) (Fig. 1B). A total volume of 100 μl was injected intradermally at the base of the tail (50 μl on each side) [14].

Liposomes were prepared by dissolving 1,2-dimyristoyl-sn-glycero-3-phosphocholine (DMPC; 850345C, Avanti Polar Lipids, Inc., Alabaster, AL) in chloroform and drying the solution to a thin lipid film in a round-bottom flask using a gentle nitrogen stream, followed by vacuum for at least 2 h [12]. The lipid film was then hydrated with 25 μg WNT16 protein (7790-WN-025/cf; R&D Systems) in 400 μl PBS prewarmed to 32 °C. The final lipid concentration was 13.2 mg/ml. Control liposomes were prepared by hydration in PBS alone. The mixture was vortexed until the solution appeared cloudy and no visible lipids remained at the bottom of the flask. Liposomes were subsequently extruded by passing the suspension 51 times through a 100-nm polycarbonate membrane (Whatman, UK) at 32 °C. After cooling to room temperature, liposomes were stored at 4 °C until use.

At days 6, 9, and 12, nine of the mice received a 30 µl intra-articular injection of WNT16 liposomes (62.5 µg/ml WNT16 in PBS) into the left hindlimb knee joint, whereas the right knee joint received a 30 µl intra-articular injection of liposomes without WNT16 diluted in PBS [12]. A second local immunization was performed at day 7, where 20 mg/ml mBSA (dissolved in PBS) was mixed 1:1 with WNT16 liposomes (125 µg/ml WNT16 in PBS), and the mice received a 30 µl intra-articular injection into the left hindlimb knee joint [12, 14]. The right knee received a 30 µl intra-articular injection of 20 mg/ml mBSA (dissolved in PBS) and liposomes without WNT16 (dissolved in PBS) at a ratio of 1:1.

To control that the second immunization induced arthritis, five of the primary immunized mice were given a 30 µl intra-articular injection of 10 mg/ml mBSA in PBS in the left hindlimb joint at day 7, whereas the right joint received a 30 µl intra-articular injection of PBS. On days 6, 9, and 12, these mice received 30 µl intra-articular injections of PBS in both hindlimbs joints.

Fourteen days after the primary immunization, the mice were euthanized using Ketador (Richter Pharma) mixed with Dexdomitor (Orion Pharma), followed by exsanguination and cervical dislocation. The hindlimbs were cleaned from the skin, separately fixed in formalin and stored in ethanol until CT analyses.

### Mouse Model of Inflammation-induced Bone Loss Caused by Toll-like Receptor 2 Activation

Inflammation-induced bone loss was initiated by subcutaneous injection of 75 μg synthetic diacylated lipopeptide, Pam2CSK4 (PAM2; InvivoGen, Toulouse, France) in 100 µl at the top of the skull [12, 17] of 6-week-old *Obl-Wnt16* (WNT16 expression driven by the rat procollagen type I alpha1 promoter) female mice and WT female littermate controls, on C57BL/6N background [13]. All experiments were carried out on female mice born from crossing a male *Obl-Wnt16* mouse with a female C57BL/6N mouse. PAM2 is an agonist of Toll-like receptor (TLR) 2. NaCl (9 mg/ml) was used as a vehicle. All injections were done under anesthesia with isoflurane (Baxter Medical AB, Kista, Sweden). Five days post-inoculation with PAM2, the mice were euthanized using Ketador (Richter Pharma) mixed with Dexdomitor (Orion Pharma), followed by exsanguination and cervical dislocation. The skull bones and femurs were dissected, separately fixed in formalin, and stored in ethanol until CT analyses. Trabecular-rich vertebral bodies were dissected, placed in RNAprotect Tissue Reagent (76 106, Qiagen), and stored at − 80 °C until total RNA extraction (Trizol Reagent, 15596018, Thermo Fisher Scientific) followed by the RNeasy Mini Kit (74116, Qiagen). To obtain cDNA, the RNA was reversed transcribed (4368814, Applied Biosystems) and real-time PCR analyses were performed using the StepOnePlus Real-Time PCR system (Thermo Fisher Scientific) using the predesigned Mm00446420_m1 assay for *Wnt16*. Relative gene expression was calculated using the 2^−∆∆Ct^ method using the ribosomal subunit 18S as internal standard.

### Mouse Model of *S*.* Aureus*-induced Arthritis

In the *S*.* aureus* arthritis experiments, food was placed at the bottom of the cage and water bottles with extra-long tips were provided to minimize discomfort for mice with inflamed paws during feeding. Pre-prepared batches of *S*. *aureus* strain LS-1, which produces Toxic Shock Syndrome Toxin 1 (TSST-1), were thawed, washed and diluted. Nine-week-old *Obl-Wnt16* female mice [13] and WT female littermate controls on C57BL/6N background were inoculated via the tail vein with 0.2 ml of *S*. *aureus* at a concentration of 5 × 10^7^ [] CFU/mouse or PBS as control.

Following inoculation, mice were weighed daily, and clinical assessments of *S*. *aureus-*induced arthritis were performed by observers blinded to the treatment group. The observers visually inspected each mouse’s wrists, ankles, fingers, and toes throughout the infection period [18]. Arthritis was defined as erythema and/or joint swelling. To assess arthritis severity, a clinical scoring system from 0 to 3 was used for each paw, as previously described [18, 19]. After eight days, the mice were euthanized using Ketador (Richter Pharma) mixed with Dexdomitor (Orion Pharma), followed by exsanguination and cervical dislocation. Lumbar vertebra 5 were dissected, fixed in formalin and stored in ethanol until CT analyses.

### Assessment of Bone Parameters

#### Determination of Periarticular Bone Mineral Density (BMD)

To determine periarticular bone loss in the AIA mouse model, both the femur and tibia were analyzed using Stratec pQCT XCT Research M software version 5.4B (Norland) at a resolution of 70 μm, as described previously [20]. Total and trabecular BMD were determined using metaphyseal scans performed 0.4 mm from the growth plate, proximally in the distal femur and distally in the proximal tibia, respectively. The trabecular bone compartment was defined as the inner 45% of the bone area.

#### High-resolution Micro-computed Tomography (μCT)

Bone morphology was analyzed using high-resolution micro-computed tomography (μCT). A SkyScan 1172 system was used for the skull and a 1275 system for the femur and fifth lumbar vertebra (L5) (Bruker MicroCT, Aartselaar, Belgium). Scanning was conducted with an X-ray tube voltage of 50 kV for the skull and 40 kV for the femur and vertebra. The current was set to 200 μA. An aluminum filter of 0.5 mm was used for the skull, and 1 mm for the femur and vertebra. The scanning angular rotation was 180° with an angular increment of 0.70°, and an isotropic voxel size of 14 μm for the skull and 7 μm for the femur and vertebra. For femur analysis, the trabecular bone proximal to the distal growth plate was selected within a conforming volume of interest, excluding cortical bone, starting 504 μm from the growth plate and extending 210 μm proximally. For skull bones, the volume of interest was 6 mm wide, extending from 2 mm anterior to the posterior tip of the frontal bone to 1 mm posterior to the anterior tip of the interparietal bone, maintaining bilateral symmetry along the interfrontal and sagittal sutures. In the vertebra, the trabecular bone in the vertebral body caudal of the pedicles was selected for analysis within a conforming volume of interest (cortical bone excluded) commencing at a distance of 7 μm caudal of the lower end of the pedicles and extending a further longitudinal distance of 245 μm in the caudal direction. Datasets were reconstructed using NRecon (version 1.6.9.8, Bruker) and further analyzed with CTAn software (version 1.20.8.0, Bruker).

### Calvarial Osteoblast Cultures and Gene Expression Analysis

Osteoblasts from CsA mice from our own inbred colony were isolated from 2–3-day-old mouse parietal bones by time sequential digestion with bacterial collagenase [21]. Cells from digestions 6–10 were used and plated at a density of 10^4^ cells/cm^2^ and incubated with or without TNF-α (50 ng/ml) for 24 h. RNA was isolated and gene expression analysis performed using TaqMan Fast Advanced Master Mix as described previously [22]. Amplifications were performed with the StepOnePlus Real-Time PCR system. β-actin was used as housekeeping gene.

### Statistical Analyses

All statistical analyses were performed using GraphPad Prism (version 10.3.1) and data are presented as mean ± standard error of the mean (SEM). Depending on the experimental design, statistical significance was assessed using either the paired or unpaired Student’s t-test, or the Mann–Whitney U test, as appropriate. To assess the effects of genotype, treatment and interaction, two-way ANOVA was used. Mouse survival rates were analyzed using the Kaplan–Meier method, with group comparisons assessed by the log-rank test. A *P* value of < 0.05 was considered statistically significant.

## Results

### TNF-α Treatment Reduces *Wnt16* mRNA Expression in Osteoblasts

To determine the direct effect of TNF-α on *Wnt16* mRNA expression, primary mouse calvarial osteoblasts were cultured for 24 h in control medium with or without TNF-α (50 ng/ml). TNF-α treatment significantly downregulated *Wnt16* mRNA expression in the osteoblasts, indicating that inflammatory signaling may directly suppress WNT16 production at the transcriptional level (Fig. 1A).

### No Effect of WNT16 Treatment in the Knee Joints on Local Bone Erosion Caused by Antigen-induced Arthritis in the Knee

Osteoclast activation at the cartilage pannus junction is a critical step in bone resorption in patients affected by inflammatory-induced arthritis such as in RA. As WNT16 is a potent inhibitor of osteoclastogenesis, we hypothesized that delivering WNT16 via liposomes, which we previously demonstrated to increase bone mass in rodent models [12], could prevent osteoclast-mediated local bone erosion in an antigen-induced arthritis disease model (Fig. 1B). Severe bone loss was observed in antigen-injected knee joints, as demonstrated by a significant reduction in total BMD in the metaphyseal regions of the distal femur and proximal tibia (Fig. 1C, D). This reduction was accompanied by a decrease in trabecular BMD in both regions (Fig. 1E, F). To assess whether WNT16 delivered via liposomes could mitigate antigen-induced bone loss, we administered liposomes, with or without WNT16, directly into antigen-injected knee joints. However, WNT16 treatment did not prevent the local antigen-induced bone erosion, when injected into the knee joint, as no protective effects were observed (Fig. 1C–F).

### Overexpression of WNT16 in Osteoblasts Does not Protect Against PAM2-induced Local or Systemic Bone Loss

**Fig. 2:**
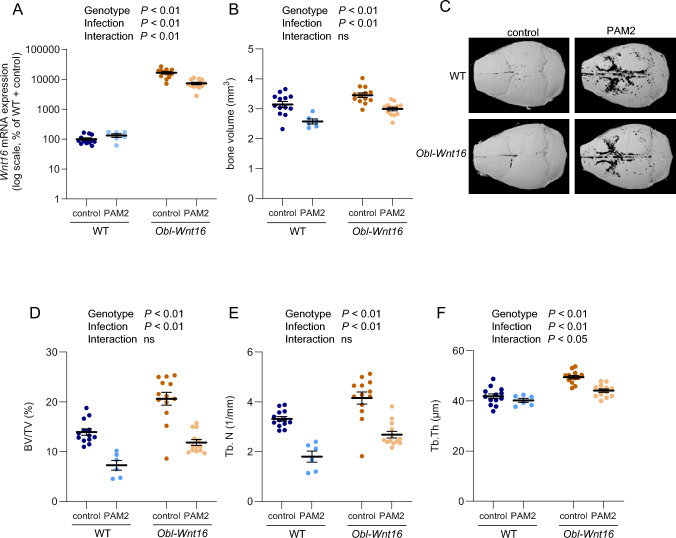
Overexpression of WNT16 does not protect against PAM2-induced bone loss. **A** *Wnt16* mRNA expression in trabecular-rich vertebral bodies. **B** Bone volume and **C** representative 3-dimensional images of calvarial bones analyzed by µCT. **D** Bone volume over total volume (BV/TV), **E** trabecular number (Tb. N), and **F** trabecular thickness (Tb. Th), in femur as measured using µCT. Data were collected from 7-week-old *Obl-Wnt16* and wild-type (WT) mice five days after injections of PAM2 or vehicle (control). Values are mean ± SEM (n = 6–13 mice per group). Statistical analyses were performed using a two-way ANOVA. ns = non-significant

To investigate whether high WNT16 expression can protect against local or systemic inflammation-induced bone loss, *Obl-Wnt16* mice, overexpressing WNT16 in osteoblasts, and their WT littermates were injected with the Toll-like receptor 2 (TLR2) agonist PAM2 at the skull. *Obl-Wnt16* mice treated with control exhibited approximately 165-fold higher *Wnt16* mRNA expression in the trabecular-rich vertebral bodies compared to WT littermate controls (Fig. 2A). Although PAM2 injection reduced *Wnt16* mRNA expression in *Obl-Wnt16* mice, the expression remained approximately 55-fold higher than in PAM2-treated WT littermates (Fig. 2A). No reduction in Wnt16 mRNA by PAM2 injection was observed in the WT littermates.

*Obl-Wnt16* mice displayed significantly higher baseline calvarial bone volume compared to WT littermate controls (Fig. 2B, C). However, PAM2 injection led to a marked reduction in calvarial bone volume in both genotypes, indicating that WNT16 overexpression did not prevent PAM2-induced local bone loss (Fig. 2B, C). A similar pattern was observed systemically. In the femur, *Obl-Wnt16* mice exhibited higher baseline trabecular bone volume fraction (BV/TV), attributable to increased trabecular number (Tb.N) and thickness (Tb.Th) (Fig. 2D–F). Nevertheless, PAM2 treatment resulted in significant bone loss in both WT and *Obl-Wnt16* mice. These findings demonstrate that although WNT16 overexpression enhances baseline bone mass, it does not protect against bone loss triggered by acute TLR2-mediated inflammation by PAM2.

### Overexpression of WNT16 in Osteoblasts Does not Protect Against *S. Aureus*-induced Systemic Bone Loss

**Fig. 3:**
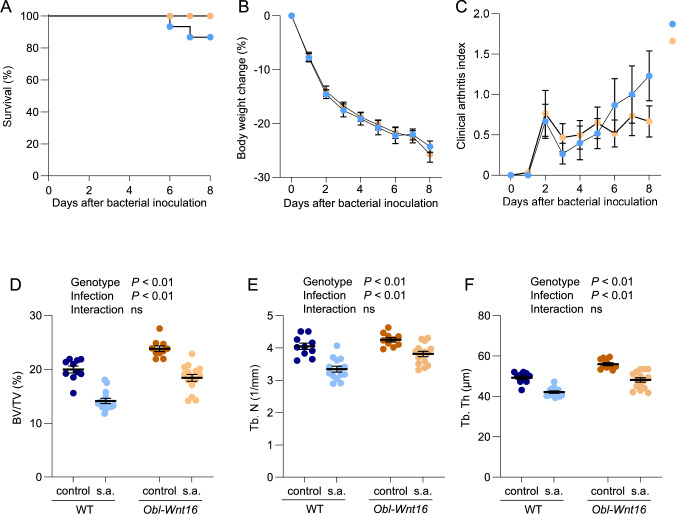
Overexpression of WNT16 does not protect against *S*. *aureus*-induced arthritis or bone loss. **A** Kaplan–Meier survival curve showing the percentage of surviving mice over time following *S*. *aureus* inoculation (n = 15 per group). Statistical comparison was performed using the log-rank test. Body weight change **B** and clinical arthritis scores **C** over the 8-day period post-inoculation. Data are presented as mean ± SEM (n = 13–15 mice per group) and were analyzed using the Mann–Whitney U test. (D-F) µCT analysis of the fifth lumbar vertebra 8 days post-inoculation, showing **D** bone volume over total volume (BV/TV), **E** trabecular number (Tb.N), and **F** trabecular thickness (Tb.Th) in PBS-treated controls and *S. aureus*-inoculated mice. Statistical analysis was performed using two-way ANOVA. s.a. = *S*. *aureus*; ns = non-significant

To investigate whether high WNT16 expression protects against *S*. *aureus-*induced systemic arthritis, *Obl-Wnt16* mice and WT littermate mice were intravenously inoculated with *S*. *aureus* and monitored for 8 days. There were no differences between *Obl-Wnt16* mice and WT littermates in survival rate or weight loss, used as an indicator of morbidity (Fig. 3A, B). Furthermore, daily clinical evaluation demonstrated no difference in clinical arthritis scores severity between *Obl-Wnt16* mice and WT littermate controls throughout the study period (Fig. 3C).

As expected, under baseline conditions, *Obl-Wnt16* mice exhibited higher vertebral trabecular BV/TV compared to WT littermate controls (Fig. 3D), driven by increased trabecular number (Tb.N; Fig. 3E) and trabecular thickness (Tb.Th; Fig. 3F). *S*. *aureus* inoculation led to a significant systemic reduction in vertebral trabecular BV/TV in both genotypes, due to a decrease in both trabecular number and trabecular thickness (Fig. 3D–F). Importantly, *Obl-Wnt16* mice were not protected from this infection-induced bone loss, indicating that WNT16 overexpression in osteoblasts does not prevent systemic bone deterioration during *S*. *aureus*-induced inflammation.

## Discussion

This study investigated the potential protective role of WNT16 in preventing inflammation-induced bone loss using multiple mouse models that reflect different types of inflammatory conditions. Although WNT16 has previously been shown to be a strong regulator of bone mass through its dual action of suppressing osteoclastogenesis and stimulating osteoblast activity [12, 13], our data demonstrate that WNT16, although in general increasing bone mass, is insufficient to mitigate inflammation-induced bone loss.

TNF-α is known to inhibit the Wnt signaling pathway in osteoblasts and bone tissue by upregulating Wnt antagonists such as DKK1 and sclerostin [23–25]. Furthermore, anti-TNF-α therapy in patients with RA has been associated with decreased sclerostin levels, further supporting a mechanistic link between TNF-α activity and Wnt signaling [26]. Consistent with this, our *in vitro* data demonstrate that TNF-α, a central pro-inflammatory cytokine implicated in bone loss during chronic inflammatory diseases [27], suppresses *Wnt16* expression in osteoblasts. These findings suggest that inflammation may impair WNT16’s protective effects on bone by limiting its levels. Consequently, enhancing WNT16 levels may represent a potential strategy to counteract the deleterious effects of TNF-α on bone mass. However, in the present study, PAM2-induced inflammation did not reduce *Wnt16* mRNA levels in the vertebrae of WT mice not overexpressing WNT16.

Although we previously demonstrated that exogenous WNT16 delivered via liposomes increases bone mass in rodent models [12], intra-articular administration of WNT16 liposome in the present antigen-induced arthritis mouse model [14] failed to prevent local periarticular bone loss. One possible explanation is that the inflammatory microenvironment in the arthritic joint may impair the biological activity or stability of exogenously administered WNT16. Alternatively, the inflammatory signaling may undermine the osteoprotective effects of WNT16. Furthermore, it is possible that the bone loss observed in the current antigen-induced arthritis mouse model is primarily driven by local mechanisms within the osteoclast bone microenvironment, which may not be adequately targeted by intra-articular administration of WNT16-loaded liposomes.

To further explore the bone-protective potential of WNT16 under inflammatory conditions, we employed a genetic approach using our recently developed mouse model with osteoblast-specific overexpression of WNT16 (*Obl-Wnt16* mice). These mice displayed higher baseline bone mass, consistent with previous reports [13]. However, *Obl-Wnt16* mice were not protected from the local or systemic bone loss following activation of TLR2 by PAM2 injection [15] or systemic infection with *S*. *aureus* [16]. Both conditions induced profound inflammation and bone loss in WT and *Obl-Wnt16* mice to a similar extent, demonstrating that enhanced WNT16 expression in osteoblasts alone is not sufficient to preserve bone under inflammatory stress. However, WNT16 has been shown to increase overall bone mass, suggesting that it may be beneficial for enhancing bone density regardless of the underlying cause of bone loss.

These results align with prior studies indicating that inflammatory cytokines not only suppress bone formation but also shift the bone remodeling balance toward resorption [2]. Although WNT16 regulates both osteoblast and osteoclast activity under homeostatic conditions, its protective effects seem to be outweighed by the strong pro-resorptive signals associated with inflammation. Notably, neither pharmacological WNT16 administration nor genetic overexpression altered disease severity or inflammatory responses in our disease models, reinforcing the idea that WNT16 operates primarily as a skeletal regulator rather than an immunomodulator.

An important consideration is that WNT16’s effects may be context-dependent. In steady-state bone remodeling, WNT16 enhances bone mass by acting on osteoblasts and inhibiting osteoclastogenesis [12, 13]. However, in inflammatory conditions characterized by high levels of DKK1, TNF-α, and other inhibitors of Wnt signaling [10], WNT16 signaling may be attenuated or insufficient. Thus, combination therapies targeting both inflammation and Wnt signaling may be necessary to effectively prevent inflammation-induced bone loss. A limitation of this study is that the effect of WNT16 overexpression was assessed only at 1–2 weeks after induction of inflammation. Whether WNT16 may protect against bone loss in the longer term remains to be determined.

In conclusion, while WNT16 enhances overall bone mass, it does not confer protection against acute inflammation-induced local or systemic bone loss*.* These findings suggest that WNT16 is not a specific therapeutic candidate for preserving bone mass during inflammation and highlights the complex interplay between inflammatory signaling and bone remodeling. Further studies are warranted to explore combinatorial approaches that might enhance WNT16 signaling or protect it from inflammatory suppression in order to understand its full osteoprotective potential.
